# Intercultural Cognitive Pragmatics as a tool for understanding autism

**DOI:** 10.3389/fnhum.2026.1729076

**Published:** 2026-02-05

**Authors:** Daniel Żuromski, Anita Pacholik-Żuromska

**Affiliations:** Institute of Information and Communication Research, Department of Cognitive Science, Nicolaus Copernicus University in Toruń, Toruń, Poland

**Keywords:** communication, autism, Cognitive Pragmatics, culture, embodiment, interaction, social cognition, Theory of Mind

## Introduction

For decades, Theory of Mind (ToM), understood as the ability to attribute beliefs, desires, and intentions to others, has served as the central framework for investigating social cognition in both typical and atypical development (Baron-Cohen, 1995; Frith and Frith, 2006). Within this paradigm, autism has often been conceptualized as a mentalizing deficit, marked by difficulty in recognizing and interpreting others' mental states. This “mindblindness” hypothesis (Baron-Cohen, 1995) has significantly shaped diagnostic approaches and guided extensive neuroscientific research into the neural correlates of ToM, particularly those activated during social reasoning tasks (Li et al., 2014).

At the same time, research conducted on Western, Educated, Industrialized, Rich, and Democratic (WEIRD) samples raised important concerns about the generalizability of psychological theories that are often presented as universal (Arnett, 2008; Henrich et al., 2010; Thalmayer et al., 2021; Barrett, 2020; Broesch et al., 2023; Muthukrishna et al., 2020; Rad et al., 2018). The ways of speaking about autism have entered public discourse and blurred into everyday thinking. In consequence scientific discourse on autism has influenced how autistic people narrate about themselves (Hacking, 2009).

With the growing influence of the socio-cultural paradigm in cognitive science, perspectives on autism have undergone significant transformation. Both interactionist and intercultural frameworks, along with attention to differences in sensory integration, have been increasingly incorporated into the field of Neurodiversity Studies (Williams, 2020; Williams et al., 2021; Proff et al., 2022; Hillary, 2020). For example, cross-neurotype and double empathy approaches argued for recognizing autistic communication as culturally situated, proposing more models that treat neurocognitive differences as expected and mutual (Milton, 2012; Stones, 2023; Marocchini, 2023).

In line with this development, and motivated by concerns of both theoretical adequacy and ethical fairness, we proposed the framework of Intercultural Cognitive Pragmatics (Żuromski et al., 2022). Our approach, briefly characterized also in this opinion, aims to strengthen the voice that, following Barrett, can be summarized as follows:

“a particular human brain in a particular human body, raised and wired in a particular culture, will produce a particular kind of mind” (Barrett, 2020, p. 55–56).

In other words, the mind is not a universal, context-free entity, but an emergent product of the interaction with the culturally structured environments. Differences in culture, social practices, and communicative norms therefore give rise to systematically different ways of perceiving, interpreting, and engaging with the social world.

## Neurocognitive characteristics and relational dynamism in autism

Although the studies of autism reveal atypical activity within the DMN (Uddin et al., 2017; Padmanabhan et al., 2017), these changes are domain-general rather than specific to mentalizing, and may reflect alterations in attentional modulation and sensory prediction (Pellicano and Burr, 2012). Within a predictive coding framework (Friston et al., 2009; Friston, 2010), the brain is a hierarchical system that minimizes prediction error. Autism, under this view, may involve increased precision weighting of sensory input, leading to heightened sensitivity and reduced reliance on prior beliefs (Lawson et al., 2014). This shift challenges the idea that autistic individuals lack mentalizing *per se*; instead, they may process the social world with different predictive dynamics, often leading to social uncertainty or withdrawal in unpredictable contexts (cf. Stoodley and Tsai, 2021). Thus, neural processes align with interactional dynamics: difficulties in social coordination may stem not from an impaired ToM system, but from a mismatch between sensory processing, predictive expectations, and cultural norms of communication.

Autistic individuals may excel in sensory detail detection and pattern recognition, but may find it more challenging to engage in rapid interactional synchrony demanded by neurotypical communicative norms, particularly when communication relies on implicit context-dependent meaning. These are not deficits of representation but variations in embodied participation. Recent research has emphasized the importance of peripersonal space (PPS) and self-location as core components of self-experience, especially in neurodivergent individuals (Noel et al., 2017). The comparative research on schizophrenia and autism spectrum disorder revealed that both exist at opposite ends of a continuum of self-other differentiation, mediated by the gradient of PPS (Noel et al., 2017). Individuals with schizophrenia tend to exhibit a shallow and unstable PPS boundary, resulting in a weakened distinction between self and other, whereas individuals with autism spectrum disorder tend to show a steep and rigid PPS boundary, leading to a stronger separation. (Noel et al. 2017) argue that a relatively shallow self–other boundary may be advantageous in certain contexts, such as facilitating empathy, while a steeper boundary may be beneficial in others, for example by supporting accurate attribution of self-generated experiences. This, in turn, may affect how communication unfolds and what is communicated.

As well as neuroscientific discoveries the studies in the field of cognitive and developmental psychology have raised serious doubts on the universality and sufficiency of ToM as a model of social cognition. For example (Gernsbacher and Yergeau 2019) document a series of empirical failures undermining the ToM-deficit hypothesis. They show that performance gaps on ToM tasks between autistic and non-autistic participants are often artifacts of linguistic complexity, unfamiliar task demands, or inconsistent measurement tools. Moreover, when presented with ecologically valid tasks characterized by explicit, informational richness, many autistic individuals perform within typical ranges or even exceed expectations. Hence, ToM-based accounts of autism may be descriptively inadequate and culturally biased, failing to capture the diversity of how people understand others.

Autism research is still predominantly conducted from a one-sided perspective, based on neurotypical standards and assumptions (Davis and Crompton, 2021). Notably, empirical studies consistently point to the relational nature of social difficulties in autism. One of the most influential contemporary approaches in autism research, the Double Empathy Problem (DEP), has challenged the traditional view that social difficulties in autism constitute a unilateral, individual cognitive deficit (Milton, 2012; Milton et al., 2022; Woods et al., 2018). Instead, it emphasizes that difficulties in “mindreading” are relational and symmetrical. While autistic individuals may struggle to understand the intentions, emotions, and behaviors of neurotypical individuals, neurotypical individuals also encounter difficulties in interpreting the behaviors of autistic people (Crompton et al., 2021). On the one hand, autistic individuals are significantly more effective at interpreting the mental states of other autistic individuals than those of neurotypicals (Williams et al., 2021). On the other hand, neurotypical individuals interpret the behaviors and mental states of autistic individuals less accurately, even though autistic people are equally expressive, albeit in different ways (Sheppard et al., 2016). While (Williams et al. 2021) examine communicative interaction in which understanding emerges through reciprocal alignment, Edey et al. (2016) focus on non-interactive, observer-based tasks that require the perceptual decoding of mental states from bodily movement alone. In the latter study, neurotypical participants are less successful in recognizing mental states from the movements of autistic individuals than from those of other neurotypicals, while autistic individuals do not exhibit a typical in-group advantage defined in terms of perceptual decoding superiority, a criterion that may privilege neurotypical movement patterns. By contrast, in the study by (Williams et al. 2021), advantages emerge within mutual interaction grounded in shared communicative norms.

Difficulties in interpreting autistic behavior among neurotypical individuals correlate with more negative social evaluations of autistic people (Alkhaldi et al., 2019). Autistic individuals must function within a world largely shaped by the norms and interpretive practices of the neurotypical majority, with autism affecting approximately one in every hundred people (Zeidan et al., 2022). Consequently, their social challenges are often framed through neurotypical standards. These cognitive problems do not represent a universal social dysfunction. Rather, they reflect different modes of experience and distinct social infrastructures that shape interactions between autistic and neurotypical individuals (Davis and Crompton, 2021). For example interactions between autistic individuals reveal a distinct, neurodivergent form of intersubjectivity. This is understood as the ability to engage in second-person interactions and co-create meaning, and is characterized by generous assumptions of shared understanding, low demands for coordination, and a high tolerance for fragmented dialogue (Heasman and Gillespie, 2019).

## The role of communication and Intercultural Cognitive Pragmatics

While the neuronal perspective is indispensable for elucidating the cognitive mechanisms underlying mental functioning, it is not, in itself, sufficient to account for the shaping of the mind (Cowley and Vallée-Tourangeau, 2013). A more comprehensive understanding of the development and functioning of the mind requires situating cognitive processes within a broader socio-cultural context (Vygotsky, 1997; Cole, 1996; Rogoff, 2003). This context should not be regarded merely as an external environment acting upon pre-existing cognitive structures, but rather as constitutive of them. As (Carpendale et al. 2016, p. 189) argue, “human minds develop within social relations. That is, social activity is constitutive of human minds.” In this light, social interaction is not ancillary to mental development but constitutes a fundamental condition of its possibility. Central to this process is communication, which not only enables the transmission of mental content but also actively co-constructs the very architecture of cognition itself. As (Carpendale and Lewis 2020, p. 195) stress, “communication is then the source of human mind.”

Even theories that strongly emphasize the role of social interaction do not always include the normative dimension of communication. For example, at the core of classical Cognitive Pragmatics (Bara and Bara, 2010; Levinson, 2025) lies the concept of social interaction, whose paradigmatic case is Tomasello's theory of shared intentionality (Tomasello, 1999, 2014, 2016). From the perspective of the DEP (Milton, 2012; Milton et al., 2022; Woods et al., 2018), this theory, despite emphasizing the socio-cultural context of cognitive development, can still be seen as a deficit-based account. This is because autistic difficulties are interpreted as limitations in the development and maintenance of joint attention and shared intentionality, which are considered biologically grounded and species-typical foundations of human social cognition (Tomasello, 2019).

In response to the need for adopting an intercultural perspective that emphasizes the role of communication we proposed Intercultural Cognitive Pragmatics (ICP), which offers an alternative interpretation of these same abilities (Wierzbicka, 2003; Kim et al., 2018; Kroupin et al., 2025; Bambini and Lecce, 2025; Taumoepeau, 2025). Communicative interactions based on joint attention and shared intentionality are here understood as mechanisms of cognitive reorganization, whose development and functional profile are modulated by local practices and cultural norms, leading to diverse developmental trajectories (Nisbett and Masuda, 2003; Shneidman et al., 2016). ICP accords with the DEP because both approaches reject deficit-based, individualistic accounts of social cognition and emphasize the relational and bidirectional nature of communicative understanding. While the DEP shows that difficulties in social understanding between autistic and neurotypical individuals are symmetrical and arise from mutual misattunement, ICP provides a pragmatic and intercultural account of the mechanisms underlying relational misunderstandings.

Our emphasis on communication builds on previous work in the field. For example (Williams 2020) and (Williams et al. 2021) explores the idea that communication between autistic and non-autistic individuals can be understood through the lens of intercultural pragmatics. She suggests that breakdowns in understanding are not due to deficits in autistic people, but arise from reciprocal differences in communicative norms, which differ widely. Rules about who may speak, what topics are appropriate, and how truth is judged, vary across societies, shaping metacognitive habits. For instance, traditional Mayan communities rely heavily on non-verbal cues, unlike WEIRD groups that prefer explicit verbal exchange. (Danziger 2010) shows that Mopan Mayan communicative norms diverge from Gricean principles: truth is judged by literal accuracy, not speaker intent, and fictional narratives are seen as untrue. Mopan speakers also avoid making claims about others' minds, often opting for silence when uncertain. Such practices teach children culturally appropriate ways of managing information (Kim et al., 2018; Lavelle, 2016, 2021).

Analogously, autistic participants often describe their own communication as direct and literal, assuming alignment between what is said and what is meant, whereas neurotypical interlocutors tend to interpret utterances in light of different pragmatic expectations (Milton, 2012; Marocchini and Baldin, 2024). In cross-neurotype interactions, this mismatch frequently leads neurotypical interlocutors to overinterpret autistic utterances, attributing ironic, impolite, or relational meanings that autistic speakers did not intend to convey, supporting an intercultural pragmatic analysis of cross-neurotype communication. As reported by participants in Marocchini and Baldin's (2024) study, such misunderstandings do not reflect deficits in communicative competence but arise from a relational misalignment in how meaning is located and interpreted, which justifies analyzing cross-neurotype communication analogously to intercultural differences within the framework of ICP.

In this context, recent empirical and theoretical work has begun to challenge the biological universalism contained in classical accounts of shared intentionality. Specifically, the canonical model of the “joint attention phenotype” characterized by gaze-following and affectively positive, triadic engagement appears to be not universally distributed, but rather predominantly observed in WEIRD populations (Bard et al., 2021). When broader definitions of shared engagement are applied, greater cross-cultural and cross-species continuity becomes visible. This suggests that what is likely universal is not a narrowly defined, biologically fixed pattern of interaction, but rather a general capacity for triadic connectedness, which can take diverse forms depending on ecological, cultural, and developmental contexts (Bard et al., 2021; Sauciuc and Persson, 2023).

In many non-WEIRD cultures, behavior is explained not through individual beliefs but through roles, relationships, and situational cues (Wierzbicka, 2003). This supports the idea of “local minds” understood as cognitive styles adapted to specific socio-cultural ecologies. Attention styles are a major source of cognitive divergence. Westerners typically focus on discrete objects, analyze their features, and infer causality from individual elements. East Asians, by contrast, attend to context, emphasize relationships, and group by similarity reflecting the analytic (Western) vs. holistic (Eastern) distinction (Nisbett, 2010; Norenzayan et al., 2007).

These neurocognitive differences align with the ICP framework, which posits that cognition is scaffolded through interaction and cultural context. From this perspective, if autism is understood as a condition marked by steep peripersonal space gradients and rigid self-boundaries, then cultural environments that reward fluid social boundaries and rely heavily on implicit social cues may pose challenges for autistic individuals. Conversely, environments that make such boundaries more explicit may enhance participation and foster mutual understanding. ICP integrates cross-cultural research, showing that different societies foster different models of social understanding. For example, in cultures characterized by an “opacity of mind,” where public commentary on mental states is normatively constrained, children tend to succeed on ToM tasks later than in WEIRD societies. Research from Samoa indicates that many children solve standard ToM tasks only around the ages of six to eight (Mayer and Riese, 2013). Moreover, the ICP framework offers a promising lens for designing therapeutic interventions. As (Noel et al. 2017) suggest, personalized multisensory training aimed at either sharpening or softening the self–other boundary may recalibrate peripersonal space gradients. For autistic individuals, this could involve carefully designed multisensory environments that gradually reduce the rigidity of self-location, enabling more flexible engagement with others.

Such body-centered interventions resonate with the ICP emphasis on participatory scaffolding, rather than isolated cognitive correction. The sociocognitive perspective aligns with ICP because it also treats social interaction as inherently communicative, shaped by abilities such as joint attention and shared intentionality. While these abilities may have biological foundations, their development is always modulated by local cultural environments, which influence and structure the pathways through which cognitive abilities emerge (Nisbett and Masuda, 2003; Shneidman et al., 2016). From this standpoint, the key question in understanding autism is not whether a person possesses an abstract ToM module, but how their participation in interactional and linguistic scaffolds supports or constrains their capacity to understand others. Building on this insight, (McGeer 2015) argues that autistic cognition should be interpreted through the lens of “scaffolded minds,” which are formed and transformed through participation in social and linguistic practices.

This approach also resonates with the work of (Hillary 2020), whose analysis highlights that intercultural communication is fundamentally reciprocal. Namely, the very concept of autism is shaped by narrative and classificatory practices, as noted by (Hacking 2009), who introduces the idea of the *looping effect*. According to this view, once categories are constructed to describe certain types of people, these categories begin to influence how individuals perceive themselves, behave, and are treated by others: How we speak about autism is a matter of culture, and this way of speaking determines how autism is perceived (Hacking, 2009). The language and framework of ToM has become so deeply embedded in autism research that it not only shapes how scientists conceptualize autistic cognition, but also how autistic individuals are expected to understand and narrate their own experiences. Such framing reinforces asymmetrical norms and overlooks the mutual nature of communicative breakdowns between neurotypes. As a result, autistic people are often depicted as communicatively incompetent, rather than simply communicating differently. Recognizing autism as embedded within cultural and communicative contexts opens the possibility for more equitable models of understanding and interaction.

## Conclusion

In this opinion paper, we advanced the position that dominant ToM-based narratives in autism research are no longer sufficient, either theoretically or ethically. Rather than treating autism as a deviation from a presumed universal model of social cognition, we argued for understanding it as a variation in how minds are shaped through communication, embodiment, and cultural participation. From this standpoint, social difficulties associated with autism are best interpreted as relational mismatches, embedded in asymmetric social structures and normative expectations, rather than as intrinsic cognitive deficits. Taking this perspective, we introduced Intercultural Cognitive Pragmatics, which, building on the groundwork established by researchers challenging the traditional pathologizing framework in studies of autistic and non-autistic communication, offers an approach for reorienting research and practice toward mutual intelligibility, participatory scaffolding, and contextual adaptation rather than individual correction.
